# Physical and Numerical Simulations for Predicting Distribution of Microstructural Features during Thermomechanical Processing of Steels

**DOI:** 10.3390/ma15051660

**Published:** 2022-02-23

**Authors:** Łukasz Poloczek, Roman Kuziak, Valeriy Pidvysots’kyy, Danuta Szeliga, Jan Kusiak, Maciej Pietrzyk

**Affiliations:** 1 Łukasiewicz Research Network-Institute for Ferrous Metallurgy, ul. K. Miarki 12, 44-100 Gliwice, Poland; roman.kuziak@imz.pl (R.K.); valeriy.pidvysotskyy@imz.pl (V.P.); 2 Department of Applied Computer Science and Modeling, Faculty of Metals Engineering and Industrial Computer Science, AGH University of Science and Technology, al. Mickiewicza 30, 30-059 Kraków, Poland; szeliga@agh.edu.pl (D.S.); kusiak@agh.edu.pl (J.K.); mpietrz@agh.edu.pl (M.P.)

**Keywords:** plastometric tests, stress relaxation tests, stochastic model, microstructure evolution, inverse analysis, identification, steel

## Abstract

The design of modern construction materials with heterogeneous microstructures requires a numerical model that can predict the distribution of microstructural features instead of average values. The accuracy and reliability of such models depend on the proper identification of the coefficients for a particular material. This work was motivated by the need for advanced experimental data to identify stochastic material models. Extensive experiments were performed to supply data to identify a model of austenite microstructure evolution in steels during hot deformation and during the interpass times between deformations. Two sets of tests were performed. The first set involved hot compressions with a nominal strain of 1. The second set involved hot compressions with lower nominal strains, followed by holding at the deformation temperature for different times. Histograms of austenite grain size after each test were measured and used in the identification procedure. The stochastic model, which was developed elsewhere, was identified. Inverse analysis with the objective function based on the distance between the measured and calculated histograms was applied. Validation of the model was performed for the experiments, which were not used in the identification. The distance between the measured and calculated histograms was determined for each test using the Bhattacharyya metric and very low values were obtained. As a case study, the model with the optimal coefficients was applied to the simulation of the selected industrial hot-forming process.

## 1. Introduction

Enhancing the strength–ductility synergy of materials has been an objective of research on structural materials for years. It was shown in many publications that a significant improvement of this synergy can be obtained by tailoring heterogeneous microstructures. Multiphase steels (advanced high strength steels (AHSS)) are a leading example of the enhanced balance between strength and ductility [1]. The special mechanical properties of multiphase steels are due to the heterogeneity in strength between their structural components [2,3]. The heterogeneity of mechanical properties may come from differences in various microstructural features [4,5]. The authors of [6] investigated compositional, microstructural and local hardness variations in a complex-phase (CP) steel. They showed that the hierarchically heterogeneous microstructure with smooth gradients of properties was promising for maintaining the balance between strength and workability. This problem has been recently investigated by many researchers. Various gradient structures with varying ferrite grain size were investigated in [7], and a significant influence of grain size distribution on mechanical properties was shown. Simultaneous improvement of strength and plasticity was obtained in [8] using a novel strategy that emphasised the importance of work hardening and thickness of gradient layer (avoiding sharp gradients characteristic for thin layers). A similar hypothesis, which combines improved local fracture resistance with smooth gradients of properties, was put forward in [9]. Although the successful control or application of chemical or microstructural heterogeneity to achieve the desired properties was achieved by some researchers (e.g., for pipeline [10], multiphase [11] and ferritic/martensitic steels [12], as well as for other fields in engineering [13]), it seems that numerical modelling can still be useful support for the design of these materials. Advanced numerical models with the capability to predict distributions of various microstructural features are needed to reach this goal.

The stochastic model, which describes the microstructure evolution during multi-step hot forming of metallic materials, was developed in [14], and it is described in [15] in the deformation part (dynamic processes) and in [16] for interpass times (static processes). The correct evaluation of coefficients in the model for a particular material has a significant influence on the accuracy and reliability of the model. This goal is usually reached by performing experimental tests and the application of inverse analysis to find optimal coefficients in the model. The inverse solution for hot forming is well described in the literature [17,18,19], which also includes the extensive discussion of the existence and uniqueness of the solution in [20]. However, all published examples of inverse analysis concern deterministic models. The situation is different for the stochastic models when measurements of histograms of the output parameters have to be considered. The mathematical background of the inverse approach to the stochastic variables is described in [15]. Furthermore, the optimal numerical parameters of the model (number of Monte Carlo points, number of bins), which provide the best convergence and accuracy of optimisation, are proposed in that paper. Beyond this, the existence and convergence of the model are discussed and various measures of the distance between the predicted and experimental histograms are analysed. The practical application of inverse analysis to the stochastic model is described in [21]. To the best of the authors’ knowledge, research on the identification of stochastic microstructure evolution models is scarce. The published papers focused on the statistical inverse problem for the identification of a non-Gaussian tensor-valued random field, e.g., in [22], or the uncertainty of inverse analysis [23]. The analysis of the published works shows that there is a need for the development of the inverse approach to the identification of the stochastic microstructure models of steels. This approach requires advanced experiments, which supply information about the heterogeneity of microstructural parameters instead of the average values. Our objective was to perform experiments that would supply reliable and accurate data for the identification of the stochastic model described in [15]. In the investigation, a thermomechanical Gleeble 3800 simulator was used to perform experiments of controlled deformation to gain the data required for the model development. The experiments allowed for identifying the kinetics of structural changes occurring during and after deformation. Using this information, further experiments were planned with water quenching to “freeze” the austenite microstructure at different stages of processes occurring in the samples. Following this, the samples were quenched to reveal austenite grain boundaries, and the measurement of grain size distribution was performed on microstructure images.

## 2. Model

The model was developed in [14] and the subsequent steps of the development are described in the literature [15,16,21]. The general idea of the model, which is based on the stochastic internal variable (dislocation density) representing microstructure evolution, is presented in [15]. In Ref. [21], the grain size was included as the second stochastic variable, and the identification of the model based on the experimental data available in the literature for medium-carbon steel was performed. In Ref. [16], the model was extended by accounting for metadynamic and static recrystallisation during interpass times, which allowed for a simulation of multi-step forming processes. The main equations of the model are repeated below to complete the present paper. The evolution of dislocation density as a stochastic internal variable is governed by the following equation [15]:

(1) $$\rho (t_{i})=\rho \left(t_{0}\right)\left[1-\xi \left(t_{i}\right)\right]+\left\{\rho \left(t_{i-1}\right)+\left[A_{1}\dot{\varepsilon }-A_{2}\rho \left(t_{i-1}\right){\dot{\varepsilon }}^{1-a_{7}}\right]\Delta t\right\}\xi \left(t_{i}\right)$$

where *t*—time; $\dot{\varepsilon }$—strain rate; *A*_1_ and *A*_2_—parameters of the model responsible for athermal storage (hardening) of dislocations and recovery, respectively; *ξ*(*t_i_*)—parameter responsible for a random character of recrystallisation; and *a*_7_—coefficient.

The coefficients in Equation (1), which are in general based on the Kocks–Estrin–Mecking (KEM) model [24,25], are defined in Table 1, where *b*—Burgers vector module; *Z*—Zener–Hollomon parameter; *l*—average free path for dislocations; *T*—temperature in K; *R*—universal gas constant; *D*—grain size; *τ*—energy per unit of dislocation length; *G*—shear modulus; and *a*_1_, *a*_2_, *a*_3_, *a*_4_, *a*_5_, *a*_9_ and *a*_10_—coefficients.

The parameter *ξ*(*t_i_*) accounts for the random character of recrystallisation, where its distribution is described by the following conditions [15]:

(2) $$\begin{array}{l} P\left[\xi \left(t_{i}\right)=0\right]=\{\begin{array}{c} p\left(t_{i}\right)\;if\;p\left(t_{i}\right)<1\\ 1\;\;otherwise \end{array}\\ P\left[\xi \left(t_{i}\right)=1\right]=1-P\left[\xi \left(t_{i}\right)=0\right] \end{array}$$

In Equation (2), *p*(*t_i_*) is a function that combines the probability that the material point recrystallises in a current time step and the present state of material:

(3) $$p\left(t_{i}\right)=a_{4}\times 10^{-10}\rho {\left(t_{i-1}\right)}^{a_{6}}\frac{3\gamma \left(t_{i}\right)\tau }{D\left(t_{i-1}\right)}\exp\left(\frac{-a_{5}}{RT}\right)\Delta t$$

where *γ*—mobile fraction of the recrystallised grain boundary area, which depends on the (already known) distribution of *ξ* in the previous step (see [26]), and *a*_4_, *a*_5_, *a*_6_ and *a*_17_—coefficients.

(4) $$\gamma \left(t_{i}\right)=1-\exp{\left\{-P\left[\xi \left(t_{i-1}\right)=0\right]-q\right\}}^{a_{8}}\left\{1-P\left[\xi \left(t_{i-1}\right)=0\right]\right\}$$

where *a*_5_, *a*_6_ and *a*_8_—coefficients, and *q*—a small number representing a nucleus of recrystallised grain, which is added to avoid a zero value of *γ*(*t_i_*) in the case of **P**[*ξ*(*t_i_*_−1_) = 0] = 0.

Details of the numerical solution are given in [15]. In [21], the grain size was introduced as a stochastic variable. The initial grain size is *D*(*t*_0_) ≡ *D*_0,_ which is a random variable. During heating, a non-uniform distribution of grain size is observed (see Section 3), and there is a tendency for large grains to begin to consume small grains [27,28]; therefore, the Weibull distribution was assumed for *D*_0_. This distribution is described by the following probability density function:

(5) $$f\left(D_{0}\right)=\frac{k}{{\bar{D}}_{0}}{\left(\frac{D_{0}}{{\bar{D}}_{0}}\right)}^{k-1}\exp\left[-{\left(\frac{D_{0}}{{\bar{D}}_{0}}\right)}^{k}\right]$$

where *k* and ${\bar{D}}_{0}$—the shape parameter and the scale parameter of the distribution, respectively.

A shape parameter of *k* = 10 was assumed in [21], which was determined based on measured grain size distributions. The scale parameter ${\bar{D}}_{0}$ of the distribution was established as the average grain size measured after preheating before deformation. Grain growth was calculated based on the fundamental research of Sellars [29], who proposed the following equation:

(6) $$D\left(t_{i}\right)={\left[D{\left(t_{i-1}\right)}^{a_{11}}+a_{12}\exp\left(\frac{a_{13}}{RT}\right)\Delta t\right]}^{\frac{1}{a_{11}}}$$

where Δ*t* = *t_i_* − *t_i_*_−1_—time step; *D*(*t_i−_*_1_) and *D*(*t*)—grain size at the beginning and the end of the time step, respectively; and *a*_11_, *a*_12_ and *a*_13_—coefficients.

During the calculation random parameter *ξ*(*t_i_*) = 0, the considered point recrystallises, and its new grain size *D*(*t_i_*) is drawn from the Gauss distribution:

(7) $$f\left[D\left(t_{i}\right)\right]=\frac{1}{\sqrt{2\pi \sigma^{2}}}\exp\left\{-\frac{{\left[D\left(t_{i}\right)-\bar{D}\left(t_{i}\right)\right]}^{2}}{2\sigma^{2}}\right\}$$

where $\bar{D}\left(t_{i}\right)$—the expected grain size value, calculated as either dynamically or statically recrystallised grain size; see details in [16].

The whole model contains 22 coefficients grouped in vector **a**. Some of these coefficients have physical meaning: *a*_3_—activation energy for self-diffusion, *a*_5_—activation energy for recrystallisation, *a*_10_—activation energy in the Zener–Hollomon parameter and *a*_13_—activation energy for grain growth. Other coefficients were introduced as the results of approximation in inverse analysis in previous research works. The application of the model to real materials and processing methods requires the identification of the model coefficients. The identification was performed in [16] using the inverse approach for the experimental data available in the literature. This identification, however, requires a special set of experimental data, including histograms of microstructure parameters at various stages of the process. These data were obtained in the experiments performed in the present work and described in Section 3.

## 3. Experiment

### 3.1. Material, Methodology and Parameters of the Tests

The material for the research was steel S355J2, which is an unalloyed, low-carbon, welded structural steel used for the hot forging and rolling of long products. As mentioned in the Introduction, two sets of tests were performed. All tests were carried out on the thermomechanical simulator Gleeble 3800. Conventional inverse analysis [19] was applied to eliminate the effects of friction and deformation heating. The first set consisted of hot uniaxial compression of φ10 × 12 mm cylindrical samples with a total strain of 1. Different strain rates and temperatures were applied. The objective was to supply data for the identification of the coefficients in the equations in Table 1 and Equations (2)–(4). The procedure of identification was based on the average dislocation density, as described in Section 3.2, and on the histograms of grain size after deformation. The parameters of the experiments in the first set of tests are given in Table 2. The second set of experiments consisted of hot uniaxial compression with lower nominal strains, followed by holding at the deformation temperature for different times *t_h_* after the completion of the recrystallisation. The time for the completion of the static recrystallisation was determined using the stress relaxation technique [30]. The objective was to supply data for the identification of static recovery and static recrystallisation parts in the model, see [19] for details The parameters of the experiments in the second set of tests are given in Table 3. As with previous experiments, two preheating temperatures were used: 1200 °C and 1100 °C. The preheating time was 120 s in all tests.

**Figure 1:**
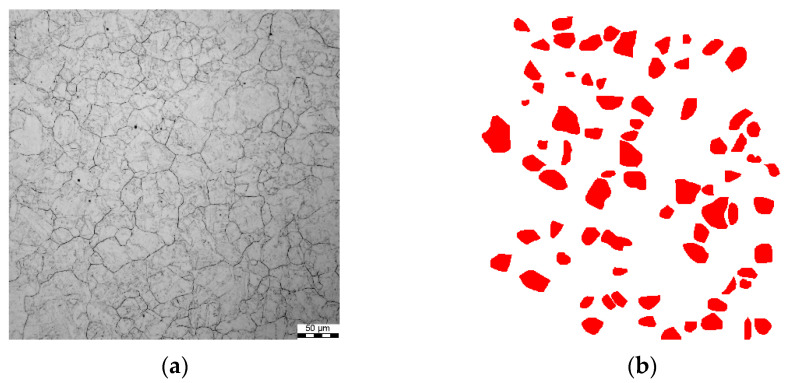
Example of the detection of austenite grain boundaries’ revealed microstructure (**a**) and the detection obtained using the MetIlo software (**b**) for sample T35.

The images used as the input for the analysis and measurement of austenite grain sizes were taken using an Olympus DSX500i light microscope at 693× magnification via the bright field technique. The equivalent diameter was used as a measure of grain size, and the measurements were performed in the central part of the sample. To reveal the grain boundaries of austenite, the samples were etched in saturated picric acid with the addition of detergent in the amount of 5 mL per 100 mL of acid at 56 °C for the time necessary to reveal the boundaries of prior austenite. This time was dependent on the deformation history. For the clear etching of grain boundaries, the material for testing was additionally subject to tempering in a Carbolite Chamber Furnace C-1600. This treatment created the desired effect, but some of the grains remained undisclosed (Figure 1a), which made further analysis much more difficult. The determination of grain size distributions and their measurement was carried out using the MetIlo program. On average, about 200 grain-size measurements were taken for each sample. In the course of the measurement, manual austenite grain correction was applied to avoid errors connected with the wrong automatic identification of boundaries. An example image of austenite grain detection after the manual correction is shown in Figure 1b.

### 3.2. Dislocation Density

**Figure 2:**
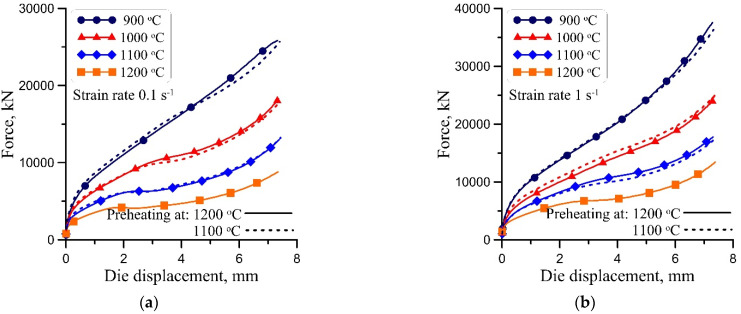

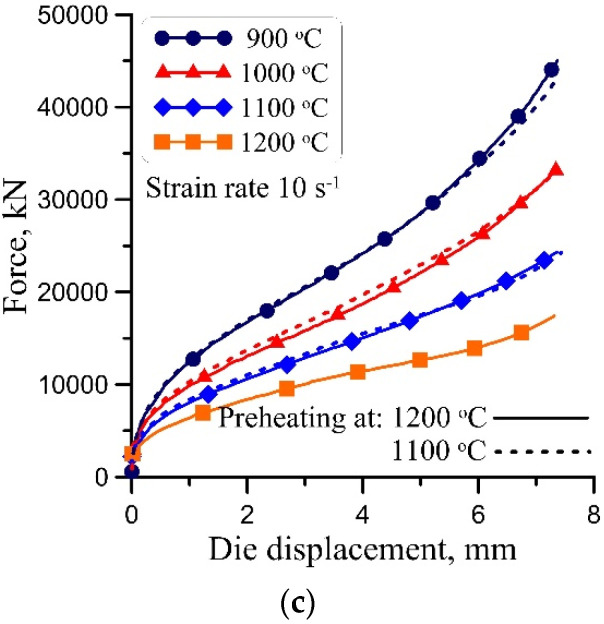
Results of force vs. die displacement in uniaxial compression tests for nominal strain rates 0.1 s^−1^ (**a**), 1 s^−1^ (**b**) and 10 s^−1^ (**c**).

Measurement of the distribution of dislocation density during hot forming is not possible for steels subject to allotropic transformations. Therefore, as it was suggested in [21], the average dislocation density calculated from the measured compression forces was used in the identification procedure. Uniaxial compression tests supplied data in the form of force vs. die displacement curves, which are shown in Figure 2. An analysis of the results showed that differences between loads for various preheating temperatures were negligible. An increase in the slope of the plots during deformation was caused by the effect of friction, which increased with an increase in the tool–workpiece contact surface. This effect was accounted for by the application of conventional inverse analysis [19].

Calculations of the flow stress vs. strain curves required accounting for the effect of heterogeneities due to the effect of friction, heat generation due to plastic work and heat transfer due to the die. The inverse approach proposed in [19] was applied. The flow stress vs. strain curves were calculated by searching for the minimum of the following objective function:

(8) $$\Phi =\sqrt{\frac{1}{Nt}\sum_{i=1}^{Nt}\left[\frac{1}{Ns}\sum_{j=1}^{Ns}{\left(\frac{F_{ij}^{m}-F_{ij}^{c}}{F_{ij}^{m}}\right)}^{2}\right]}$$

where *Nt*—number of tests; *Ns*—number of load measurement sampling points in one test; and $F_{ij}^{m}$ and $F_{ij}^{c}$—measured and calculated forces, respectively.

The flow stress determined using inverse analysis was used to calculate the average dislocation density as a function of time. The changes in the average dislocation density were calculated using the following equation based on the KEM [24,25] model:

(9) $$\rho_{m}\left(t\right)={\left[\frac{\sigma_{m}\left(t\right)}{\alpha MbG}\right]}^{2}$$

where *σ_m_*—flow stress calculated from the measured compression forces, *b*—length of the Burgers vector, *G*—shear modulus, *M*—the Taylor factor (≈3 for an FCC structure) and *α*—a constant called the Taylor coefficient hereafter. Following the discussion in [31], *α* = 0.5 was assumed in the present work.

The accuracy of the calculations of the average dislocation density (*ρ_m_*) depends on the correctness of the evaluation of the shear modulus *G*, which is a function of temperature. The dependence of elastic modulus *E* and shear modulus *G* on the temperature was investigated by several studies but, unfortunately, there are large discrepancies between the published data (cf. [32,33,34]). The authors of [34] recapitulated the results for various steels and proposed the following approximation function for the relation *E* = *E*(*T*).

(10) $$E=E_{0}\left\{b_{1}\exp\left[-{\left(\frac{T-b_{2}}{b_{3}}\right)}^{2}\right]+c_{1}\exp\left[-{\left(\frac{T-c_{2}}{c_{3}}\right)}^{2}\right]\right\}$$

where *E*_0_—elastic modulus at room temperature.

The coefficients *b*_1_, *b*_2_, *b*_3_, *c*_1_, *c*_2_ and *c*_3_ that appear in Equation (10) were determined in [34] and they are given in Table 4. In the present work, to make the results closer to the conditions of hot forming of steels, coefficient *c*_2_ was slightly modified [21].

**Figure 3:**
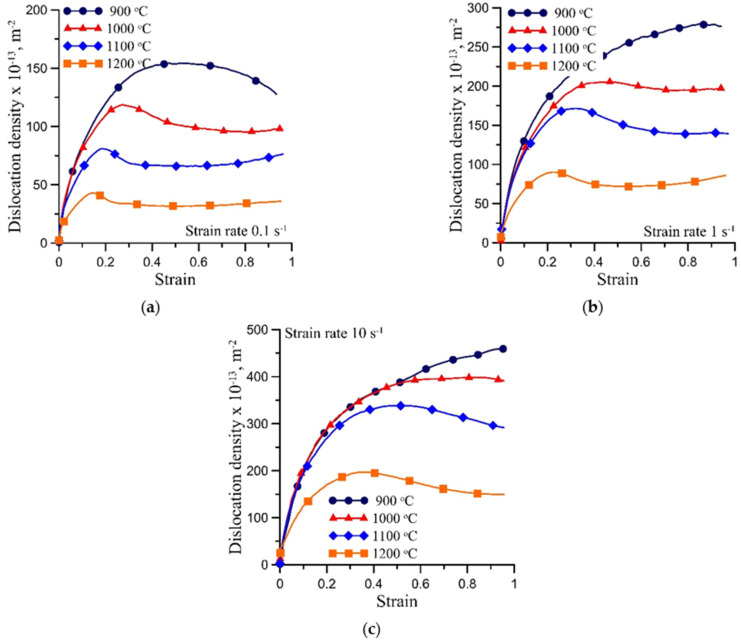
Average dislocation density vs. logarithmic strain calculated from the measurements of forces during compression for nominal strain rates 0.1 s^−1^ (**a**), 1 s^−1^ (**b**) and 10 s^−1^ (**c**).

Plots of the average dislocation density vs. logarithmic strain, which were calculated from the measurements of forces during compression, are shown in Figure 3. The logarithmic strain is defined as *ε* = ln(*h*_1_/*h*), where *h*_1_ and *h*—height of the sample before the compression and during the compression, respectively. The effect of the dynamic recrystallisation of the evolution of the dislocation density is clearly visible in this figure. The dislocation density data were used in the identification of the model.

### 3.3. Histograms of Grain Size after Preheating (Prior to Deformation)

**Figure 4:**
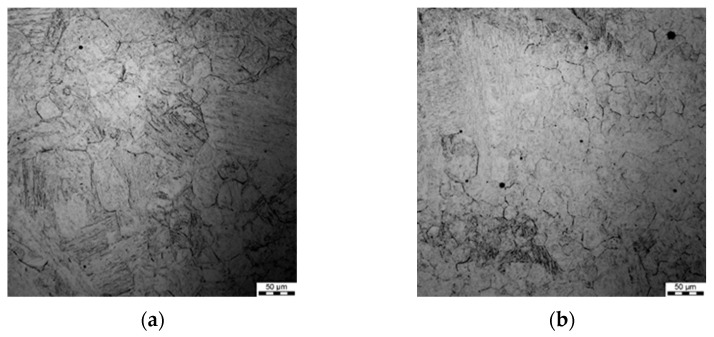
Micrographs of samples after preheating for 120 s at 1200 °C (**a**) and 1100 °C (**b**).

**Figure 5:**
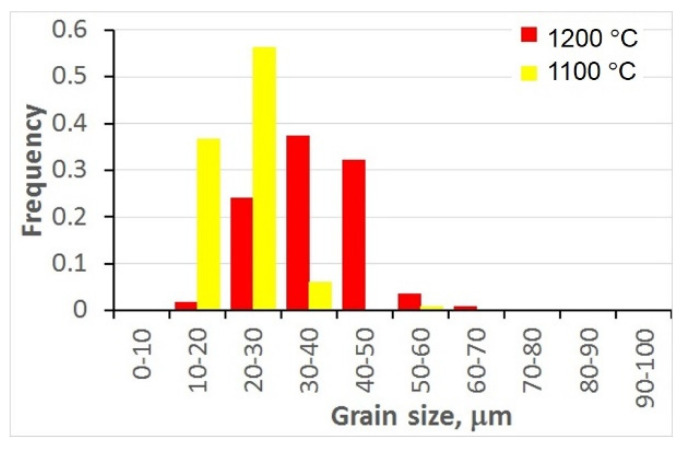
Histograms of grain size prior to deformation for the preheating temperatures 1200 °C and 1100 °C.

After each test, the samples were etched and austenite grain boundaries were revealed. Micrographs of the samples after preheating are shown in Figure 4. Histograms of the grain size prior to deformation for the two preheating temperatures (*T_p_*) are shown in Figure 5. The average grain size for the histograms was 36.5 μm and 22.5 μm for *T_p_* = 1200 °C and *T_p_* = 1100 °C, respectively.

### 3.4. Grain Size after Hot Deformation (Dynamic Recrystallisation)

**Figure 6:**
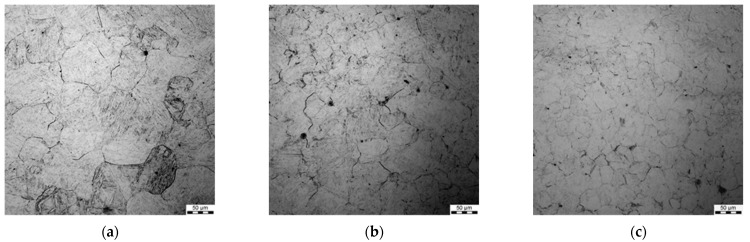
Micrographs after deformation at 1200 °C with strain rates 0.1 s^−1^ (**a**), 1 s^−1^ (**b**) and 10 s^−1^ (**c**), preheating temperature 1200 °C.

**Figure 7:**
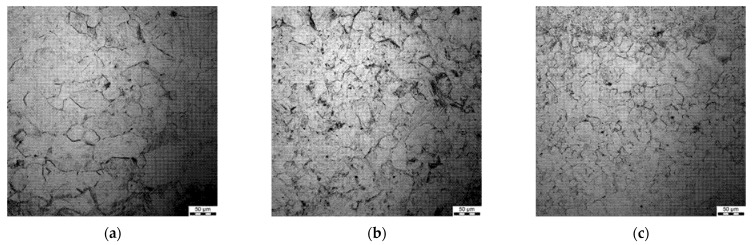
Micrographs after deformation at 1100 °C with strain rates 0.1 s^−1^ (**a**), 1 s^−1^ (**b**) and 10 s^−1^ (**c**), preheating temperature 1200 °C.

**Figure 8:**
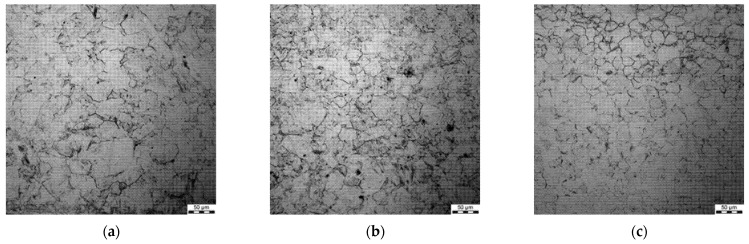
Micrographs after deformation at 1000 °C with strain rates 0.1 s^−1^ (**a**), 1 s^−1^ (**b**) and 10 s^−1^ (**c**), preheating temperature 1200 °C.

**Figure 9:**
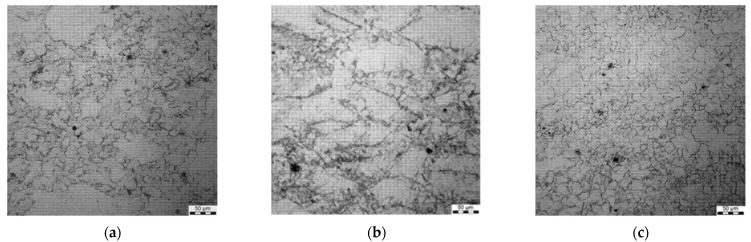
Micrographs after deformation at 900 °C with strain rates 0.1 s^−1^ (**a**), 1 s^−1^ (**b**) and 10 s^−1^ (**c**), preheating temperature 1200 °C.

All the samples were subjected to metallographic investigations; selected microstructures of samples preheated at 1200 °C followed by deformation are shown in Figure 6, Figure 7, Figure 8 and Figure 9.

Generally, a substantial spread of the measured grain diameters was observed at a higher deformation temperature (1200 °C vs. 1100 °C). Moreover, the spread was larger for lower strain rates. For the applied deformation temperatures, dynamic recrystallisation was initiated in the steels, which is coherent with the fact that grain size in the steady-state regime of the deformation depends on the Zner–Hollomon parameter.

### 3.5. Grain Size during Interpass Times (Metadynamic and Static Recrystallisation)

**Figure 10:**
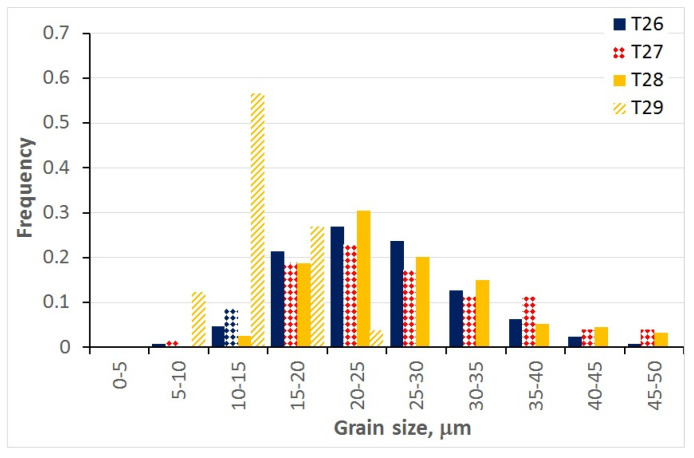
Examples of histograms measured at the end of selected static recrystallisation tests.

Selected examples of grain size histograms measured at various times after the end of deformation are shown in Figure 10. The parameters of the tests are given in Table 3. It can be seen that a substantial spread of the distribution of equivalent diameter was obtained. This can be attributed to the spread of the initial size distribution, as well as to the partially stochastic nature of the static recrystallisation phenomenon. As opposed to the samples subject to dynamic recrystallisation, the histograms of equivalent diameter were strain-dependent.

## 4. Identification and Validation of the Model

The identification procedure is described in [16]. Inverse analysis was applied to determined the coefficients **a** in the model. The aim of this analysis was to find the optimal values of these coefficients by searching for the minimum of the following objective function:

(11) $$\Phi (a)=\Phi_{\rho }(a)+\Phi_{DDRX}(a)+\Phi_{DSRX}(a)$$

where

(12) $$\Phi_{\rho }(a)=w_{\rho }\sum_{i=1}^{Ntc}d\left(\rho_{ci}\left(a\right),\rho_{mi}\right)$$

(13) $$\Phi_{DDRX}(a)=w_{D}\sum_{i=1}^{Ntc}d\left(H_{ci}\left(a\right),H_{mi}\right)$$

(14) $$\Phi_{DSRX}(a)=w_{D}\sum_{i=1}^{Nti}d\left(H_{ci}\left(a\right),H_{mi}\right)$$

Here, *ρ_c_*(**a**)—expected average value of dislocation density calculated for the model coefficients **a**, *ρ_m_*—average dislocation density determined from the compression tests (see Section 3.2), *H_c_*(**a**)—distribution of grain size calculated for the model using the coefficients **a**, *H_m_*—distribution of grain size measured in the experiments, *Ntc*—number of compression tests, *Nti*—number of measurements of histograms during interpass times and *w_ρ_* and *w_D_*—weighted coefficients.

In Equation (12), *d*(*ρ_ci_*(**a**),*ρ_mi_*) was defined as the mean square root error (MSRE) between the measured and the calculated average dislocation density in the *i*th experiment:

(15) $$d\left(\rho_{ci}\left(a\right),\rho_{mi}\right)=\frac{1}{Ns}\sqrt{\sum_{j=1}^{Ns}{\left(\frac{\rho_{cij}\left(a\right)-\rho_{mij}}{\rho_{mij}}\right)}^{2}}$$

where *Ns*—number of sampling points for measurements of average dislocation density in the *i*th test.

In parts of the objective Equations (13) and (14), *d*(*H_ci_*(**a**),*H_mi_*) was defined as the distance between the measured and the calculated histograms of grain size in the *i*th experiment. Following the analysis in [15], the Bhattacharyya [35] metric was used to calculate this distance. This metric is calculated as follows:

(16) $$d\left(H_{c},H_{m}\right)=-\log\sum_{i=1}^{n}\sqrt{P_{m}\left(i\right)P_{c}\left(i\right)}$$

where *n*—number of histogram bins; *H_m_* and *H_c_*—measured and calculated histograms, respectively; and

(17) $$P_{k}\left(i\right)=\frac{H_{k}\left(i\right)}{\sum_{i=1}^{n}H_{k}\left(i\right)}$$

The experiments were divided into two sets. Hot deformation tests (T1, T3, T5, T7, T13, T15, T19 and T20) and static recrystallisation tests (T25, T28, T32 and T35) were used in the identification procedure, and the remaining tests were used for the validation of the model. The coefficients in the model determined for the objective Equation (11) are given in Table 5.

## 5. Model Validation

**Figure 11:**
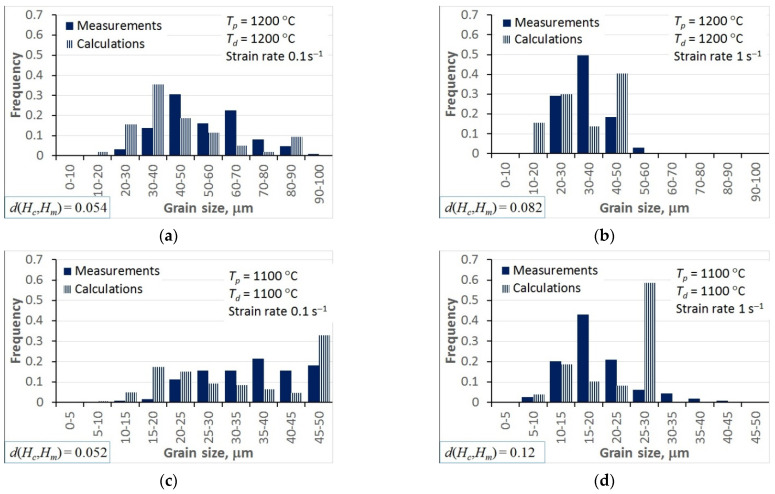
Selected examples of the comparison between measured and calculated histograms of the grain size after hot deformation tests: T1 (**a**), T2 (**b**), T13 (**c**) and T14 (**d**).

**Figure 12:**
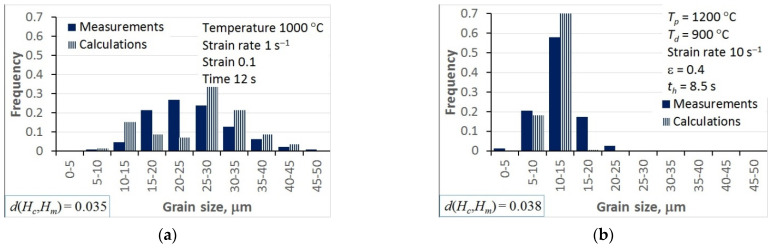
Selected examples of the comparison between measured and calculated histograms of the grain size measured at the end of the static recrystallisation tests T25 (**a**) and T29 (**b**).

Predictions of the model with the optimal coefficients were compared with the experiments, and selected results are presented in Figure 11. Some selected results for static recrystallisation tests are shown in Figure 12. The Bhattacharaya distance is given in the bottom-left corner of the plot. The visual comparison of the measured and calculated histograms confirmed the qualitatively good predictive capability of the model.

**Figure 13:**
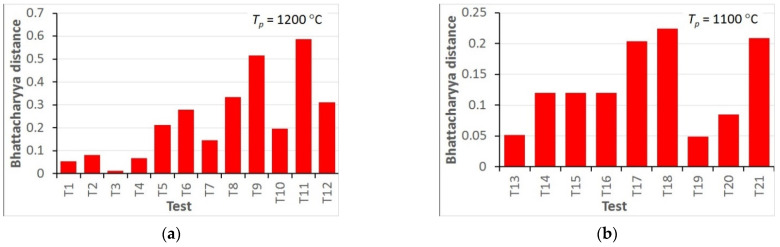
Bhattacharyya distance calculated for all hot deformation tests for preheating temperatures 1200 °C (**a**) and 1100 °C (**b**).

**Figure 14:**
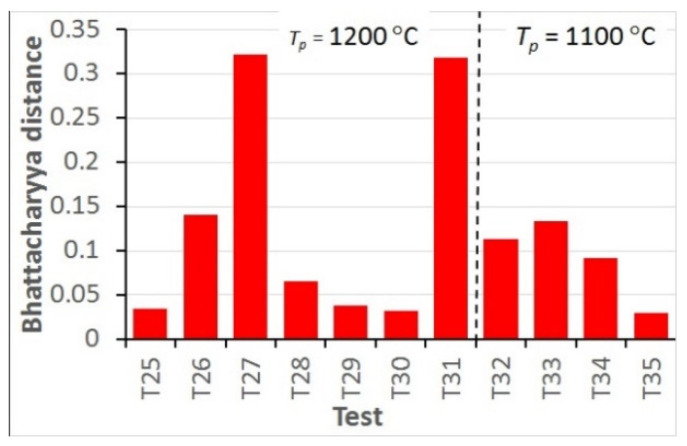
Bhattacharyya distance calculated for all static recrystallisation tests.

The accuracy of the model can be better evaluated using a quantitative comparison of the distance between the measured and calculated histograms defined by Equation (16). The values of the Bhattacharyya distance for all hot deformation tests are shown in Figure 13 and for all static recrystallisation tests in Figure 14.

**Figure 15:**
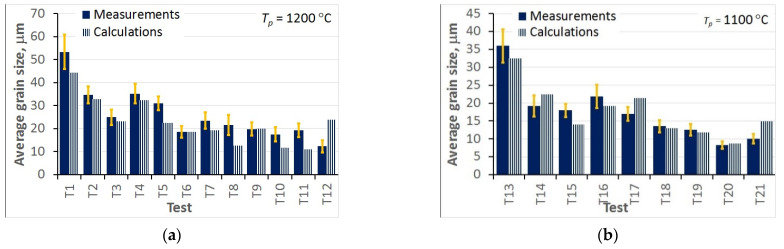
Comparison of the measured and calculated average grain sizes for all hot deformation tests for preheating temperatures 1200 °C (**a**) and 1100 °C (**b**).

**Figure 16:**
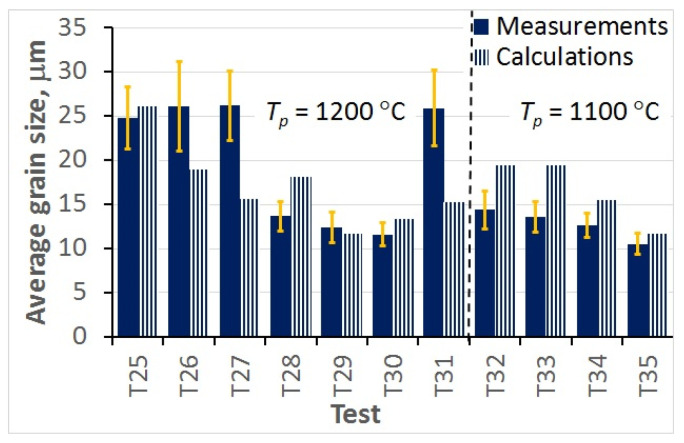
Comparison of the measured and calculated average grain sizes for all static recrystallisation tests.

The comparison between the measured and calculated average grain sizes was an additional evaluation of the accuracy of the model. These results are shown in Figure 15 for all hot deformation tests and in Figure 16 for all static recrystallisation tests. The comparisons of the measured and calculated grain sizes generated conclusions that qualitatively agreed with the observations based on the Bhattacharyya distance shown in Figure 13 and Figure 14.

## 6. Discussion of Results

Advanced stochastic models, which predict the distribution of microstructural features instead of average values, can describe the heterogeneity of microstructures of modern multiphase steels. The accuracy and reliability of such models depend on the proper identification of coefficients for a particular material. The need for advanced experimental data for the identification of the stochastic material model was a motivation for this work.

Extensive experiments were performed to supply data for the identification of the model of austenite microstructure evolution in steels during multi-step hot deformation and during interpass times between deformations. The applied experimental methodology allowed for the precise identification and quantitative description of the processes involved in austenite microstructure restoration in the process of deformation using controlled deformation parameters. The crucial condition to generate an accurate model is to precisely perform austenite boundary etching. Boundaries that are not clearly etched need to be manually reconstructed, which may lead to a significant error in the process of generation of austenite grain size distribution histograms.

The analysis of the optimisation procedure in the inverse solution showed that the Bhattacharyya metric was an efficient and reliable measure of the distance between the measured and calculated histograms. A good convergence of the optimisation was observed, and reasonably low values of the Bhattacharyya metric were obtained for all the tests. The results confirmed the good accuracy and reliability of the inverse stochastic approach. It can be assumed that a Bhattacharyya metric below 0.3 is acceptable for the comparison of microstructures. The predictions of the model with optimal coefficients were in good agreement with the measurements. The Bhattacharyya metric was below 0.2 for the experiments used in the identification, and below 0.33 for the experiments used in the validation. The value of 0.3 was slightly exceeded in a few tests, which may have been due to experimental errors. In the tests with the largest Bhattacharyya metrics, the standard deviations in the experiments were large (e.g., T1, T26, T31). This problem will be the subject of further research.

In the hot deformation experiments, better accuracy was obtained for tests at higher temperatures and lower strain rates (e.g., T1–T4) in which dynamic recrystallisation dominated. In the static recrystallisation experiments, better accuracy was obtained for the tests with larger strains and at higher temperatures (e.g., T28–T30) in which recrystallisation was faster.

## 7. Case Study

**Figure 17:**
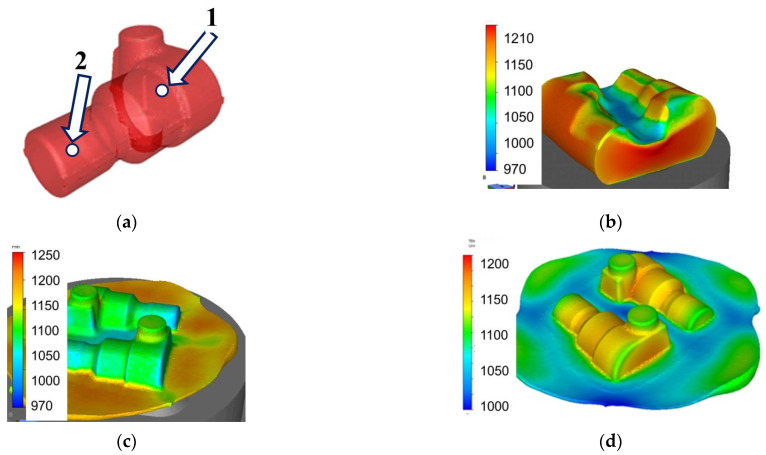
View of the hot-forged part investigated in the present work (**a**), distributions of temperature after stage 1 (**b**), stage 2 (**c**) and stage 3 (**d**) of the forging process.

Simulations of hot forging industrial processes were performed to demonstrate the predictive capabilities of the stochastic model. Forge 3D finite element (FE) software was used to simulate the thermal and mechanical phenomena during deformation and during interpass times. Forge is a computational package dedicated to the simulation of metal forming processes. The software consists of a loosely coupled set of programs, bound by a common launcher user interface. The package also consists of a database with material models. Forge 3D offers automatic adaptive remeshing, which is the main advantage of this software. The post-processing application takes a project input file and uses its data for estimating results and showing them to the user through the graphical user interface (GUI). Three-step forging of the part shown in Figure 17a was considered. The details of the industrial forging process for this part are given in [36], where more information on the application of Forge 3D to simulations of hot forging can be found. The results of FE simulations in the form of temperature distribution after subsequent stages of forging are presented in Figure 17b–d.

**Figure 18:**
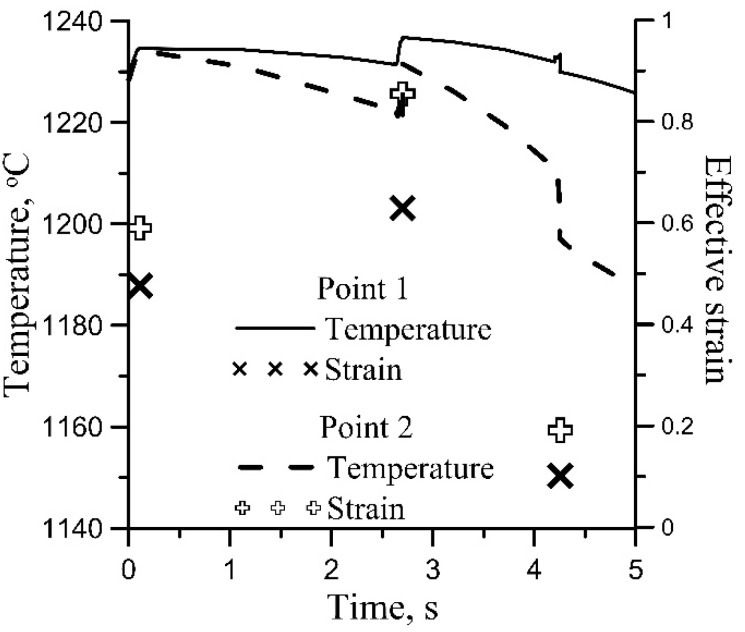
Time–temperature–deformation histories for points 1 and 2 in Figure 17a.

The solution of the stochastic evolution Equation (1) can be performed for each Gauss point of the FE mesh, accounting for the current local temperatures and strain rates. However, such detailed information is usually not needed, and it is satisfactory if the histograms are calculated in the selected critical points in the forging. IT was done by using the “sensors” in the Forge software. Forge allows for selecting a few points (sensors) in the volume of the forging in which the time–temperature–deformation history is recorded. This history was used to solve Equation (1) and to calculate histograms of dislocation density and grain size in the selected points. The results for points 1 and 2 in Figure 17a are presented in Figure 18. These points represented the massive part and thin part of the forging and were subjected to different time–temperature–deformation histories.

**Figure 19:**
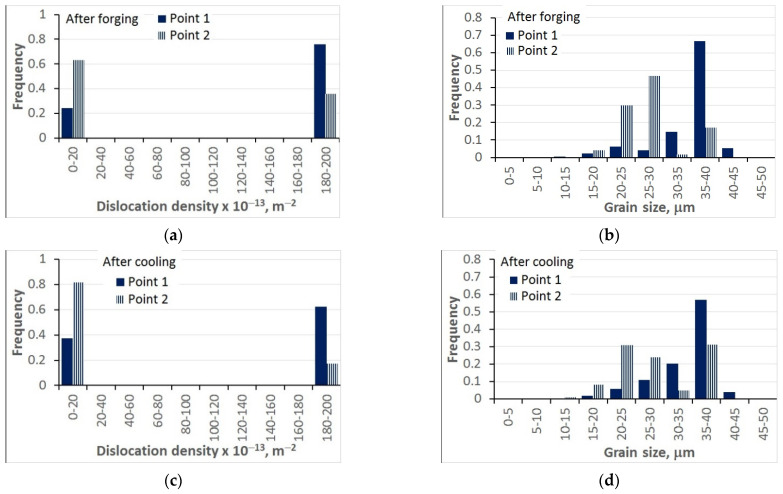
Calculated distributions of dislocation density (**a**,**c**) and grain size (**b**,**d**) at the end of the three-stage forging (**a**,**b**) and after cooling to 820 °C with a rate of 25 °C/s (**c**,**d**).

A simulation of the manufacturing cycle composed of three-stage forging followed by accelerated cooling with a rate of 25 °C/s to the temperature 820 °C was performed. The calculated distributions of dislocation density and grain size at the end of forging and after cooling to 820 °C are shown in Figure 19. The analysis of the results confirmed the model’s capability to supply quantitative information about the distribution of microstructural parameters in the complex industrial hot-forming processes. The predictions were in qualitative agreement with the authors’ knowledge about the forging process. For point 2, which was subjected to larger deformations, the dynamic recrystallisation was faster and the grain size was smaller. This tendency was maintained during cooling. Finer grains and a larger static recrystallisation volume fraction were obtained for point 2.

The representation of the dislocation density histograms was not convincing. This model was based on the assumption that the bins in the histograms were of equal length. Consequently, the majority of the material either had a very large dislocation density in the last bin or, after recrystallisation, had very low dislocation density and was located in the first bin. The volume fraction of the material with intermediate dislocation densities was negligible. In the future, the authors will change the model and introduce a different length of the bins.

## 8. Conclusions

The identification of the stochastic model of hot forming for metallic materials requires experimental data, which supplies information about distributions of microstructural parameters for different process conditions. Tests composed of hot deformation and holding after deformation for different times were performed. The following conclusions were drawn:

- The objective function in the inverse analysis was formulated as a distance between the measured and the calculated histograms. It was shown that using the Bhattacharyya metric as a measure of this distance was a very efficient approach. The results confirmed the good accuracy and reliability of the inverse stochastic approach. The predictions of the model with optimal coefficients agreed with the measurements. The Bhattacharyya metric was below 0.2 for the experiments used in the identification and below 0.33 for the experiments used in the validation, which is a reasonable accuracy when steel microstructures are compared.
- In the hot deformation experiments, better accuracy was obtained for the tests at higher temperatures and lower strain rates (e.g., T1–T4) in which dynamic recrystallisation dominated. In the static recrystallisation experiments, better accuracy was obtained for the tests with larger strain (T28–T30) in which recrystallisation was faster.
- It was observed that the accuracy of the model identification was directly dependent on the quality of the etching of austenite grain boundaries. Since, typically, not all boundaries were clearly etched, a manual reconstruction of the missing boundaries segments needed to be done, which could have resulted in significant errors.
- The lower accuracy of the developed model for low strains could be connected with a significant overlapping of the recovery and recrystallisation processes, which needs the improved methodology of the separation of their effect in plastometric studies. This will be the subject for future activities.
- The model with optimal coefficients was applied to the simulation of the three-stage hot-forging process. The predictions of the model were in qualitative agreement with the authors’ knowledge regarding hot forging, and the extensive predictive capabilities of the model were confirmed. Dislocation density (when the recrystallisation of austenite is not completed) and grain size at the beginning of phase transformation have a strong influence on the kinetics of these transformations. Thus, calculated distributions of the microstructural features can be used as a starting point for stochastic modelling of the phase transformation and prediction of histograms of phase composition in the final product. This problem will be the scope of the authors’ future research.

## Figures and Tables

**Table 1:** Relationships describing the coefficients in Equation (1).

Hardening	$A_{1}=\frac{1}{bl}$$\;\mathrm{where}\;l=a_{1}Z^{-a_{9}}$, $Z=\dot{\varepsilon }\exp\left(\frac{a_{10}}{RT}\right)$
Recovery	$A_{2}=a_{2}\exp\left(\frac{-a_{3}}{RT}\right)$

**Table 2:** Parameters of hot deformation tests for the preheating temperatures of 1200 °C and 1100 °C. The samples were water quenched directly after deformation.

*T_p_* = 1200 °C, *ε* = 1			*T_p_* = 1100 °C, *ε* = 1		
Test	*T_d_*, °C	$\dot{\varepsilon }$, *S*^−1^	Test	*T_d_*, °C	$\dot{\varepsilon }$, *S*^−1^
T1	1200	0.1	T16	1100	0.1
T2	1200	1	T17	1100	1
T3	1200	10	T18	1100	10
T4	1100	0.1	T19	1000	0.1
T5	1100	1	T20	1000	1
T6	1100	10	T21	1000	10
T7	1000	0.1	T22	900	0.1
T8	1000	1	T23	900	1
T9	1000	10	T24	900	10
T10	900	0.1			
T11	900	1			
T12	900	10			

**Table 3:** Parameters of static recrystallisation tests for the preheating temperatures of 1200 °C and 1100 °C.

*T_p_* = 1200 °C					*T_p_* = 1100 °C				
Test	*T_d_*, °C	$\dot{\varepsilon }$, *S*^−1^	*ε*	*t_h_*, s	Test	*T_d_*, °C	$\dot{\varepsilon }$, *S*^−1^	*ε*	*t_h_*, s
T25	1000	1	0.1	12	T32	1000	1	0.2	8
T26	1000	10	0.2	7	T33	1000	10	0.2	7
T27	900	1	0.2	42	T34	900	1	0.2	38
T28	1000	1	0.4	1.7	T35	900	10	0.2	17
T29	900	10	0.4	8.5					
T30	900	1	0.4	12					
T31	900	10	0.2	44					

**Table 4:** Coefficients in Equation (10) for the investigated steel.

*b*_1_	*b*_2_, °C	*b*_3_, °C	*c*_1_	*c*_2_, °C	*c*_3_, °C
0.1871	−12.96	132	0.9199	181.6	670

**Table 5:** Optimal coefficients in the stochastic model.

*a*_1_	*a*_2_	*a*_3_	*a*_4_	*a*_5_	*a*_6_	*a*_7_
0.000846	302256	334,936	1.145	290,476	1.7925	0.295
*a*_8_	*a*_9_	*a*_10_	*a*_11_	*a*_12_	*a*_13_	*a*_14_
0.786	0.28	3,740,945	4.3	5 × 10^21^	410,000	67,484
*a*_15_	*a*_16_	*a*_17_	*a*_18_	*a*_19_	*a*_20_	*a*_21_
0.274	20.91	0.5	45	0.29	0.19	6430
